# The role of nutrition and gut microbiome in childhood brain development and behavior

**DOI:** 10.3389/fnut.2025.1590172

**Published:** 2025-06-09

**Authors:** Yue Jiang, Yuanyuan Li

**Affiliations:** Department of Nutrition and Food Science, University of Maryland, College Park, MD, United States

**Keywords:** brain development, cognitive functions, behavior, maternal nutrition, gut microbiome, bioactive compounds

## Abstract

The intricate relationship between nutrition, the gut microbiome, and brain development has garnered significant attention in recent years, concerning its implications for child behavior and cognitive function. The gut-brain axis mediates this relationship through microbial modulation of inflammation, neuroactive compounds, and blood–brain barrier integrity, particularly during prenatal and early postnatal periods. Healthy dietary patterns such as whole foods, high-fiber foods, and minimally processed foods play a crucial role in shaping the gut microbiota, promoting microbial diversity and overall gut health. As a result, a balanced and diverse microbiome supports healthy brain function and development. Furthermore, disruptions in gut microbiota composition have been linked to various neurodevelopmental disorders in children, including autism spectrum disorder, attention deficit hyperactivity disorder, and anxiety. By integrating findings from animal models, clinical trials, and epidemiological studies, this review summarizes current advances on how early-life nutrition and gut microbiota interaction influence brain development and childhood behaviors. Ultimately, this paper underscores the potential for dietary interventions to promote optimal neurodevelopmental health and address behavioral issues in children.

## 1 Introduction

Early childhood, particularly the first 1,000 days, is marked by rapid growth and developmental milestones, making it a foundational phase that significantly influences cognitive, emotional, and physical well-being throughout life (1). This stage is also crucial for addressing the risk of neuropsychiatric conditions (2). During this timeframe, the brain experiences exponential growth in volume and the formation of neuronal pathways. Brain volume doubles in the first year and grows an additional 15% in the second year, reaching approximately 80% of its adult volume. Both white and gray matter volumes increase gradually over time (2). By using *in vivo* magnetic resonance spectroscopy, researchers studied the developmental profiles of six metabolites in five brain regions (3). They found that concentrations of N-acetyl-aspartate, creatine, and glutamate increased rapidly from birth to 3 months, coinciding with the rapid growth of axons and the formation of synapses. Notably, even in the first three months, before the introduction of solid foods, infants receive essential nutrients exclusively through breast milk or formula, both of which play a crucial role in shaping early brain development (1). This highlights the first 3 months of life as a critical period of rapid metabolite changes in brain development.

Neurodevelopment is crucial for long-term health and is significantly influenced by early-life experiences. During this stage, much of brain development is primarily guided by genetic programming (4). Also, emerging evidence highlights the role of epigenetic mechanisms, heritable changes in gene expression that do not alter the DNA sequence, in mediating the effects of environmental factors such as nutrition, stress, and microbial exposure. The epigenetic mechanism enables the reprogramming of the epigenome in response to external stimuli (5). Nutrition during early childhood is particularly crucial for providing the necessary nutrients to support healthy brain growth and development. It acts as a major epigenetic regulator (6). *In utero* development from conception is also crucial for the formation of the fetal central nervous system (CNS). It is essential to prioritize nutrients that can enhance neural health and brain development during early developmental stages, including macro-and micronutrients like calories, protein, fatty acids, iron, zinc, iodine, and choline for overall brain function (6). Conversely, malnutrition or poor dietary practices during these critical stages may lead to developmental delays, cognitive impairments, and an increased risk of chronic diseases later in life (6).

In utero, maternal nutrition plays an important role in supporting fetal development and influencing long-term health outcomes in the offspring, as key processes like neural tube formation and neurogenesis occur in utero and are sensitive to nutrient deficiencies. Inadequate intake of certain key nutrients during pregnancy may lead to impaired fetal programming, resulting in adverse health outcomes in adulthood (7). While nutrition in the first three months, especially through breastfeeding, supports rapid brain growth and functional maturation, it cannot fully reverse prenatal deficits (8). Some studies indicated that higher-quality maternal dietary patterns are associated with improved visual–spatial skills in early childhood and enhanced intelligence and executive function in mid-childhood (9). For example, studies have shown a positive association between specific dietary components like seafood (10), fruits (11), and nuts (12) and childhood cognitive functions and intelligence quotient (IQ). Although maternal influences on offspring neurodevelopment are well-established, emerging evidence suggests that paternal factors also contribute to early brain development and neurodevelopmental disorders. Paternal health, age, diet, and exposure to environmental toxins can all influence sperm quality and epigenetic programming (13).

**Figure 1:**
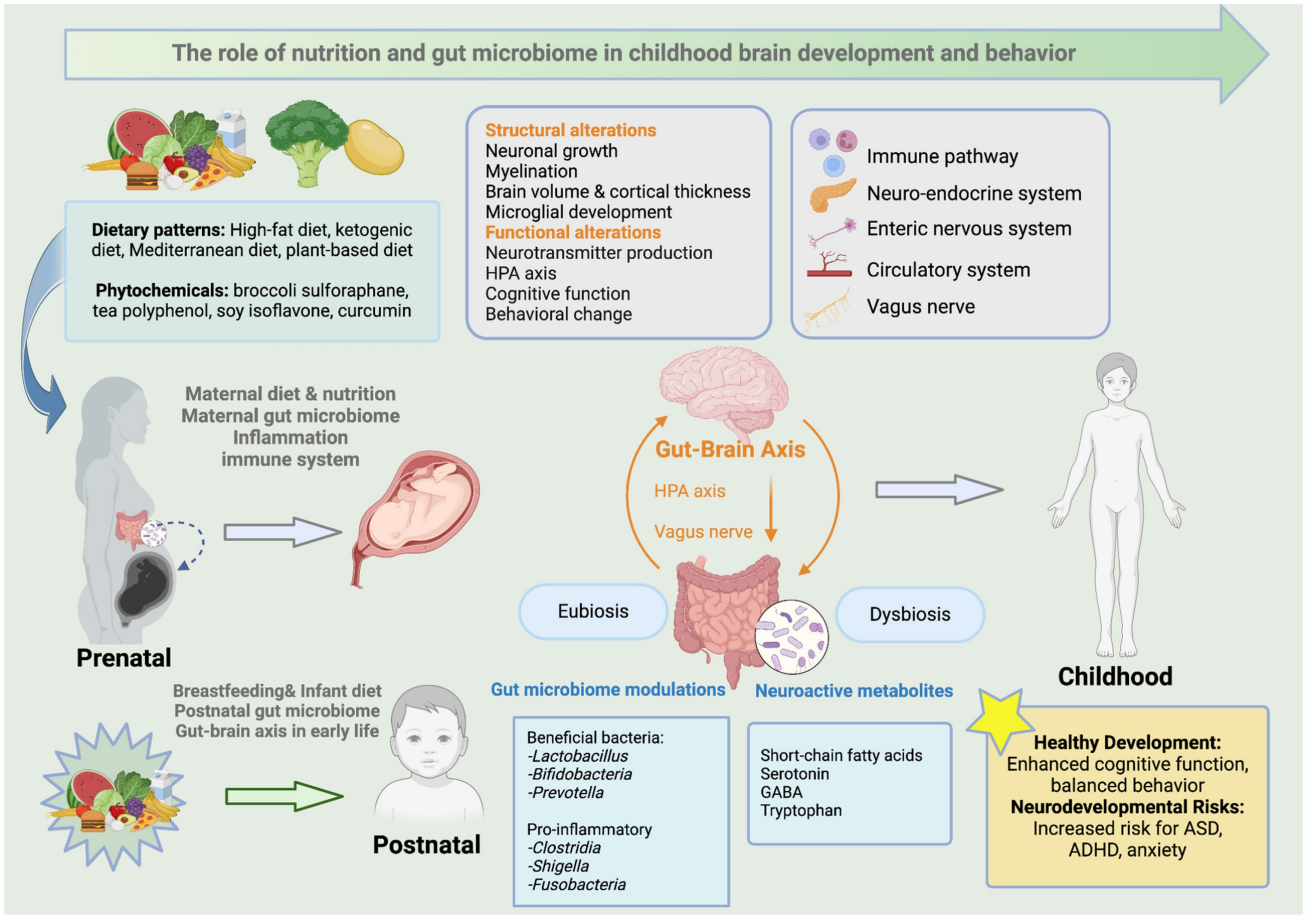
The role of early-life nutrition and gut microbiome in childhood brain development and behavior. This figure illustrates the impacts of early-life dietary patterns and phytochemicals on the gut microbiome composition in mothers and their offspring. This change modulates brain structure and functions through neuroactive metabolites that interact with the GBA via various pathways such as the immune, neuroendocrine, and vagus nerve. It further links microbial alterations to neurodevelopmental outcomes in children. Created with BioRender.com.

The gut microbiome is a vast community of trillions of microorganisms residing in the gastrointestinal (GI) tract. This complex ecosystem plays a critical role in human health, influencing various body functions and contributing to overall well-being. Fetal gut colonization may initiate in utero, facilitated by unique microbial communities in the placenta and amniotic fluid (14). The initial colonization of the microbiome coincides with neurodevelopment at the same critical developmental windows that are vulnerable to disruption (15). The gut-brain axis (GBA) refers to the bidirectional communication network between the GI tract and the CNS. Recent research suggests that microbes and their metabolic by-products play an active role in regulating early brain development. Neurodevelopment, including myelination, neurogenesis, and microglia activation, depends on the gut microbial composition, indicating that initial colonization and microbiota maturation can have long-lasting effects on mental well-being later in life (16). However, disturbances during this crucial developmental period can adversely affect brain function, potentially leading to various neurodevelopmental and neuropsychiatric disorders (17). For example, early-life gut dysbiosis caused by maternal and postnatal factors, such as diet, stress, and infection, may lead to systemic and neuroinflammation, ultimately resulting in abnormal brain development (17). As shown in Figure 1, early-life dietary patterns and phytochemicals influence the gut microbiome composition in mothers and their offspring, further altering neuroactive metabolites that interact with the GBA via immune, neuroendocrine, and vagus nerve pathways. These interactions contribute to structural and functional brain alterations, impacting cognitive function and behavioral patterns. In summary, a balanced gut microbiome, or eubiosis, supports healthy neurodevelopment, whereas an unbalanced gut microbiome, or dysbiosis, is associated with an increased risk of neurodevelopmental disorders.

Considering the significance of early-life nutrition and the gut microbiome during the critical developmental window, it is essential to explore their roles in the GBA and the potential effects on brain development and childhood behavior. Understanding how the gut microbiome influences brain development and function through various pathways offers valuable insights into the interplay between early-life diets, microbes, cognitive functions, and behavioral well-being. It also directs future nutrition guidelines that can reduce the risk of behavioral disorders and improve mental health in children.

## 2 Early brain development and neurological health

### 2.1 Early brain development and influential factors

Brain development is a protracted process that begins as early as the third week of gestation and continues from childhood to adulthood (18). During the prenatal stage, the CNS undergoes rapid growth and differentiation, including neural tube formation, the proliferation of neural cells, and the early stages of synaptogenesis, synapse pruning, and myelination (19). By birth, the brain has established its basic structure, setting the stage for continuous growth postnatally. This dynamic and essential process lays the foundation for brain development and cognitive, emotional, and social functioning throughout life. Brain development during this period is characterized by significant synaptogenesis and myelination, which enhance neural connectivity and function. Brain gray matter, which includes neuronal cell bodies, and white matter composed of nerve fibers, continue to grow and develop throughout childhood and into later adolescence (18). Brain development from early childhood to early adulthood supports the sensory, language, social, and emotional systems critical for cognitive and behavioral functioning (19).

Although genetic factors are the primary contributors to early brain development, environmental factors, such as nutritional status and exposure to toxins, also play key roles in this process (20). Some factors include maternal exposure to viral infections, drugs, and heavy metals, which may be linked to impaired brain development as well as cognitive and behavioral disorders (21). Maternal diabetes significantly increases the risk of brain malformations in the fetus. For example, two specific cerebral malformations strongly associated with maternal diabetes are holoprosencephaly and caudal regression syndrome (20). Despite the metabolic conditions of mothers, maternal nutritional status is essential for fetal brain development. Maternal obesity has been linked to impaired neurodevelopment and the increased risk of psychiatric disorders, such as Attention Deficit Hyperactivity Disorder (ADHD) (22) and Autism Spectrum Disorder (ASD) in children (23). Likewise, a study focused on adults whose mothers were exposed to undernutrition during the Dutch Hunger Winter revealed that they performed more poorly on selective attention tasks, highlighting the long-term impact of prenatal nutrition on cognitive functioning later in life (24). Fetal growth restriction (FGR) is a condition where a fetus does not grow to its expected size during pregnancy, resulting from a combination of maternal, fetal, and placental factors. Specifically, maternal causes of FGR include poor nutrition, obesity, hypertension, smoking, and chronic diseases (25). FGR has been shown to significantly impair normal brain development, leading to reduced brain volume, head circumference, gray matter volume, total number of neurons, and myelin content, all of which are associated with brain functional deficits (25, 26).

### 2.2 Mental and behavioral health disorders

Early brain development builds the foundation for cognition and behavior throughout the lifetime. Disturbances, such as nutritional deficiencies, environmental toxins, stress, and infections, during this critical developmental window may lead to mental or behavioral disorders (27). Mental health disorders (MHD) are prevalent among children and adolescents and have been steadily increasing in the past decades (28). Specifically, MHD includes emotional conditions like obsessive-compulsive disorder, anxiety, and depression, as well as behavioral disorders such as oppositional defiant disorder (ODD), conduct disorder (CD), and ADHD, often accompanied by speech or language delays and other developmental disabilities such as ASD (29). Between 2016 and 2019, the most commonly diagnosed mental disorders among U. S. children aged 3–17 were ADHD, anxiety, behavior problems, and depression (30). These emotional and behavioral disorders often co-occur and bring negative impacts on daily functioning and overall well-being, leading to increased risk of morbidity and early mortality in children. Moreover, childhood emotional and behavioral disorders bring short-term and long-term complications later in their personal and professional lives (31). Disruptive behavior problems in parents, including ADHD, ODD, and CD, have been associated with an increased risk of abusive behavior toward their offspring (32). Additionally, research suggests that early onset of MHDs can increase the risk of developing chronic mental health issues in adulthood, such as severe depression and substance abuse (33). Notably, about 50% of adults with mental disorders report symptom onset during adolescence. Overall, mental and behavioral disorders are among the leading noncommunicable diseases affecting the health of children and young adults (34).

### 2.3 IQ and cognitive function

Early brain development, characterized by the overproduction and subsequent pruning of synapses, is crucial for establishing cognitive functions such as attention, memory, thinking, learning, and perception (35). The morphology of the frontal, mesial prefrontal, temporal, and occipital regions is linked to later motor abilities, while features of the posterior parietal regions correlate with subsequent language development (36). Additionally, the temporal and occipital regions are correlated with future cognitive performance. Similarly, the properties of white-matter microstructure are moderately associated with working memory performance in one-year-old children (37). Also, the increase in white-matter volume reflects the improvements in motor performance, sensory, and auditory information (38). These studies collectively suggest that the integrity of brain structure facilitates communication between different brain regions, thereby contributing to the development of early cognitive abilities. IQ is a composite measure that reflects a range of cognitive abilities, including memory, problem-solving, logical reasoning, and verbal skills. It is influenced by both genetic and environmental factors (39). Studies indicate that changes in brain volume during early childhood and adolescence influence IQ performance (40). Additionally, cerebral volume has been shown to be positively associated with IQ level, particularly with cortical gray matter volume in the prefrontal region of the brain (41). Nevertheless, environmental factors play significant roles in influencing intelligence during childhood development, including the home environment and parenting practices, access to education and learning resources, and healthcare and nutritional support.

## 3 Gut microbiome in early brain development

### 3.1 Roles of the gut microbiome in early development

It has been widely acknowledged that the gut microbiome shares a symbiotic relationship with the host. Disruption in the gut microbiome is associated with an increased risk of various diseases, including inflammatory bowel disorders (42), metabolic diseases such as obesity and diabetes (43), and neurological disorders. In infants, the gut microbiome has a significant influence on the development of crucial physiological functions necessary for infant growth (44). During infancy, the gut microbiome is essential for various biological functions, including the digestion and metabolism of colostrum, breast milk, formula, and transitional foods (44).

Early childhood is a critical period for establishing a gut microbiome community, which exhibits high intra-and inter-individual variability due to a combination of factors, including birth mode, feeding, and environmental exposures (45). In terms of the number of species and types, the gut microbiome composition is primarily influenced by diet, especially during the first three years of life. This period featured significant dietary pattern transitions, including breastfeeding or formula feeding, weaning, and the gradual introduction of various solid foods (46). In the first six months, breast milk also plays a foundational role in establishing the infant gut microbiome by promoting the growth of beneficial bacteria such as *Bifidobacterium* and *Lactobacillus* (47, 48).

In recent years, the association between maternal diet and infant gut microbiome composition has gained intensive attention. Primary dietary elements, such as fiber, fats, and proteins, are strongly linked to distinct gut bacteria clusters in both mothers and newborns, highlighting the significant role of maternal nutrition in shaping the early gut microbiome (49). Maternal diets also impact the diversity and bacterial composition of breast milk, primarily by influencing the profile of human milk oligosaccharides (HMOs), which are essential to the health benefits of breast milk (50, 51). HMOs predominantly affect the fat composition and immunomodulatory components in breast milk, which in turn impact the infant’s gut microbiome and immune development. The influence of maternal diets on the infant gut microbiome is further supported by animal studies, which demonstrate that maternal dietary patterns can change the offspring gut microbiome as early as the pregnancy period (52–54).

### 3.2 The gut-brain axis (GBA)

The GBA describes the bidirectional communication between the gut microbiome and the brain, mediated through neural, immune, and endocrine pathways. This process is facilitated by the production of neuroactive compounds, metabolites, and hormones regulated or produced by the gut microbiome (55, 56). The GBA mechanism involves several systems, including the CNS, autonomic nervous system (ANS), enteric nervous system (ENS), hypothalamic pituitary adrenal (HPA) axis, immune system, gut microbes, and their metabolites (55). The parasympathetic and sympathetic branches of ANS transmit afferent signals from the gut ENS to the CNS. Conversely, the efferent signals are transmitted from the CNS to the ENS in the intestinal wall (57). The HPA axis transmits information from the brain stem to the gut via the vagus nerve and sensory neurons (58). Together, these systems form a dynamic and interconnected network that governs physiological functions and responses to environmental stimuli, including stress, digestion, and immune regulation.

The gut microbiome can alter the level of neurotransmitter precursors or regulate the production of various neurotransmitters (59). For instance, *γ*-aminobutyric acid (GABA) can be produced by *Lactobacilli*, *Bifidobacteria*, and *Bacteroidetes*, while serotonin synthesis is supported by *E. coli* and specific *Lactobacillus* strains (60). Brain function depends on neurotransmitter-driven signals between neurons and glial cells. Excitatory neurotransmitters include glutamate, acetylcholine, and dopamine, while GABA, glycine, and serotonin are inhibitory neurotransmitters. These neurotransmitters are essential for bowel physiological regulation, mood, and the neuroendocrine system (61). Growing evidence indicates that neurotransmitters, amino acids, and microbial metabolites, such as short-chain fatty acids (SCFAs), can enter portal circulation, influencing the immune system, metabolism, and neurons in the ENS or signaling to the brain via the vagus nerve (56). The vagus nerve enables the two-way connection between the brain and the gut microbiome by exchanging signals produced by microbial metabolites, inflammatory responses, or neuroendocrine cells influenced by the gut microbiota (62).

An imbalanced gut microbiome community may profoundly interrupt the relationship between the gut and brain, leading to mental and behavioral disorders and gastrointestinal diseases (63, 64). As a result, abnormal levels of neurotransmitters can contribute to the development of neurological and psychological disorders (65). Additionally, the disrupted gut microbiome affects the gut barrier integrity, further impairing the transmission of signaling molecules such as neurotransmitters (66). SCFAs have neuroactive properties that play a key role in maintaining and regulating the integrity of the gut and the blood–brain barrier (BBB) (67).

The substantial comorbidity between psychiatric/neurological disorders and GI conditions implies the potential involvement of bidirectional communication between the gut and the CNS (68). Dysbiosis enables pathogenic microbes, bacterial metabolites, and luminal content to pass freely through the bloodstream to the CNS, leading to potential neurological conditions. For instance, these elements can impair the BBB, trigger microglial activation, and induce damage in neuronal cells (68). Increased bacterial metabolites and inflammatory cytokines in the gut and the BBB lead to neuroinflammation, contributing to brain immunological dysfunctions (69).

Germ-free (GF) animals have been widely used to examine the roles of the gut microbiome in brain functions. Studies on GF animals have shown the causal relationship between the gut microbiota and the regulation of brain function and behavior (70). These studies revealed that abnormal brain development, marked by deficits in neuronal plasticity, impaired neuroprotection, altered neurotransmission, disrupted myelination, and behavioral abnormalities, is closely associated with inadequate colonization of the early-life gut microbiome and altered GBA (71, 72). For example, GF mice demonstrated a higher incidence of anxiety-like behaviors, as well as social and cognitive conditions such as memory dysfunction and increased motor activity, than conventionally-raised mice (16, 73).

Changes in the gut microbiome due to other factors such as antibiotic use, dietary habits, and infections are also linked to the development and progression of mental and behavioral disorders (74). Early-life antibiotic use increases the abundance of *Escherichia, Staphylococcus*, and *Clostridioides,* but reduces the abundance of important SCFA producers (75). In animal studies, antibiotic treatment can cause dysbiosis of the gut microbiome, resulting in impaired spatial memory and increased anxiety-like behavior in rats (70, 76). Disruptions to the gut microbiota due to antibiotics can cause significant changes in the immune system, driving it toward a more pro-inflammatory state of the CNS (77). Research has shown that early-life antibiotic use induced changes in gut microbial diversity that directly cause neuroinflammation and alter the structure and function of the brain, contributing to mental and behavioral disorders in children (59, 78). Moreover, psychosocial factors such as insecure mother-infant attachment have also been associated with increased antibiotic use in infancy, suggesting that caregiving environments may indirectly influence gut microbial development and neurobehavioral outcomes (79).

### 3.3 Roles of the gut microbiome in childhood neurological conditions

Studies have shown that gut microbiota can influence various aspects of brain function, including learning, memory, social behavior, and behaviors related to anxiety and depression (80). The causal association between the gut microbial profile and mental and behavioral issues has been supported by fecal microbiome transplantation (FMT) in GF animal models (68). The GF recipient mice that underwent FMT from mice with behavioral issues developed psychiatric symptoms similar to those of the host. In addition, approximately 70–90% of patients who suffer from psychiatric disorders have comorbid GI symptoms such as constipation and diarrhea (81, 82).

#### 3.3.1 Autism spectrum disorder (ASD)

ASD is a developmental disability characterized by difficulties in social interaction and restricted, repetitive behaviors or activities. In 2020, approximately one in 36 children was diagnosed with ASD (83). Symptoms may appear in early childhood and can vary in severity, affecting language development, sensory processing, and behaviors. The etiology of ASD can be genetic and non-genetic factors (84). Genetic factors contribute to about 60% of the ASD cases, while the remaining cases may be influenced by environmental factors such as maternal nutritional and metabolic status, infection during pregnancy, and toxin exposure (84).

Several studies have reported that early-life gut dysbiosis is associated with childhood ASD (85, 86), characterized by decreased levels of SCFAs and microbial producers (82). These gut microbiome dysfunctions are closely linked to the severity of core symptoms of ASD (87). In animal models and clinical cases, ASD has been linked to elevated levels of bacterial strains such as *Bilophila*, *Clostridium*, *Dorea*, and *Lactobacillus*, along with a reduced abundance of *Blautia* (88). In comparison to healthy children, ASD patients have *Firmicutes* and *Bacteroidetes* as the dominant taxa, with *Proteobacteria* and *Actinobacteria* being the least abundant (88). Specific microbial alterations include a decrease in *Bifidobacterium*, *Blautia*, *Prevotella*, *Veillonella*, and *Turicibacter*, accompanied by an increase in *Lactobacillus*, *Bacteroides*, *Desulfovibrio*, and *Clostridium.* In particular, *Clostridium* is the most commonly found bacteria enriched in ASD patients, possibly due to its ability to produce exotoxins, propionate, and p-cresol, which may worsen the severity of ASD symptoms (89, 90).

#### 3.3.2 Attention deficit hyperactivity disorder (ADHD)

ADHD, another common neurodevelopmental disorder, is characterized by symptoms such as hyperactivity, inattention, and impulsivity. Some children may experience anxiety, depression, ODD, and CD alongside ADHD (91). Based on data from 2022, around 7 million (11.4%) U. S. children have been diagnosed with ADHD (91). ADHD can be caused by genetics, non-inheritable factors, and complex interplay. Genetic predisposition plays a significant role, with about 70% heritability (92). Despite the gene variants, prenatal and perinatal influences, including maternal smoking, poor nutrition, premature birth, and exposure to environmental toxins, are associated with an increased risk of ADHD in children (92).

ADHD is also associated with alterations in gut microbiome composition caused by early-life food intake (93). Studies have found that children with a higher adherence to a healthy diet were less likely to develop ADHD, while the Western dietary pattern increased the risk (94). Moreover, essential nutrients such as omega-3 fatty acids, iron, zinc, and polyphenols play a crucial role in mitigating ADHD risk (95). Given that pediatric patients with ADHD often experience GI discomfort, the impacts of diet on both gut health and neurodevelopment may provide a key link between nutrition and ADHD symptoms (96). Microbiome studies in ADHD patients have shown decreased diversity and reduced levels of short-chain fatty acids (SCFAs), which are critical for neuroimmune signaling (59). Research suggests that within the gut microbiome of individuals with ADHD, the phylum *Actinobacteria*, particularly the genus *Bifidobacterium*, is present in significantly higher levels compared to those without ADHD, indicating a potential role of *Actinobacteria* in the development of ADHD symptoms (97, 98). Another study reported that ADHD patients had a significantly lower abundance of the genus *Faecalibacterium*, which has shown a negative association with the severity of ADHD symptoms (99). Besides, a study identified a significant increase in bacterial genes that encode cyclohexadienyl dehydratase in ADHD cases compared to controls (98). This enzyme is crucial for the synthesis of phenylalanine, a precursor to dopamine, suggesting a potential microbial influence on dopamine-related pathways in ADHD (98).

However, the results on the impacts of the gut microbiota on ADHD show mixed findings. Some studies have shown no significant differences in the diversity of the gut microbiome between ADHD patients and healthy populations (98–101). The bacterial genera *Clostridiales* and *Porphyromonadaceae* have been reported to have either elevated or reduced levels across different studies. The genus *Bifidobacterium* also showed high variability, with some studies showing an increase in ADHD subjects. In contrast, others report reductions, especially in species such as *B. longum* and *B. adolescentis* in infant samples (102). These inconsistencies reflect the complex roles of the gut microbiome in ADHD.

#### 3.3.3 Depression and anxiety

Depression and anxiety are increasingly recognized as significant mental health concerns among children. Although cognitive behavioral therapy is the most common therapy for anxiety and depression in children, studies have shown that a healthy lifestyle, such as a balanced diet and physical activity, can help manage these symptoms (103).

Animal models demonstrated the effects of the gut microbiome on modulating the neurological features of depression. FMT from depressed patients to GF mice showed the impact on initiating the symptoms of depression (104, 105). Studies found that the most affected bacterial phyla include *Firmicutes*, *Actinobacteria*, and *Bacteroidetes*, with a notable increase in the *Bacteroidetes/Firmicutes* ratio in depressed individuals (105). This imbalance was marked by an increased presence of the genus *Bacteroides* and a decrease in the genera *Blautia*, *Faecalibacterium*, and *Coprococcus* (106, 107).

Preclinical studies supported the effectiveness of microbial treatment, such as probiotics supplementation and FMT, in the prevention or treatment of the onset and progression of depression (108). In animal models, the relevant symptoms in GF-mice with depression, anxiety, and a highly active HPA were attenuated by supplementing the mice with *Bifidobacterium infantis* and *Lactobacillus,* along with the modulation of GABA receptors by the vagus nerve (109, 110). One study found that children with depression showed increased bacterial richness and distinct *β*-diversity compared to healthy controls (111). Pro-inflammatory genera such as *Streptococcus* was elevated in the depression group, while anti-inflammatory genus such as *Faecalibacterium* was diminished, leading to alterations in the production of immunomodulatory metabolites (111). This disrupted gut microbiome community correlates with the increased level of pro-inflammatory bacteria but a decreased level of butyrate-producing bacteria (106).

#### 3.3.4 Cognition

In addition to its influence on mental health, the gut microbiome also plays a significant role in cognitive function, particularly during critical developmental periods (108). Disturbances in microbiome development may negatively affect the development of cognitive functions (112). A study of school-aged children revealed a strong connection between full-scale IQ (FSIQ) and the gut microbiome composition. Higher FSIQ scores were linked to a greater gut microbiome *α*-diversity and specific taxa, including *Prevotella*, *Dialister*, *Sutterella*, *Ruminococcus callidus*, and *Bacteroides uniformis* (113). In another study, in children older than 18 months, several microbial species were notably enriched in those with higher cognitive function scores, including *Alistipes obesi*, *Asaccharobacter celatus,* and several SCFA-producing species, such as *Eubacterium eligens* and *Faecalibacterium prausnitzii* (114). Interestingly, the same study discovered that different gut microbial species were related to various cognitive functions. For example, *Bifidobacterium pseudocatenulatum*, *Blautia wexlerae*, and *Eubacterium eligens* were key predictors of expressive language, while *Roseburia faecis*, *Streptococcus salivarius*, and *Fusicatenibacter saccharivorans* may be linked to gross motor skills (114). Similarly, another longitudinal study analyzed bacterial composition data from infants aged 3–6 months and found that a higher abundance of *Bacteroides*, accompanied by a lower presence of *Escherichia/Shigella* and *Bifidobacterium*, was negatively associated with fine motor skills. Additionally, the increased abundance of *Lachnospiraceae* and *Clostridiales* taxa and reduced *Bacteroides* were linked to poorer communication, personal, and social skills at 3 years of age (115). Surprisingly, several studies have found that higher alpha diversity in infancy was associated with poorer performance on early learning composite, visual reception, and expressive language skills (116, 117). This finding contradicts the commonly accepted view that greater microbial diversity is beneficial, possibly due to reduced dominance of neurodevelopment-supporting bacteria. These findings suggest that a specific microbial composition, rather than greater diversity alone, may be critical for supporting early cognitive development (117).

## 4 Prenatal and early-life nutrition in brain development

In the prenatal period, environmental factors, including the maternal diet, toxin exposure, and stress, can significantly impact fetal brain development. Among these, maternal nutrition during pregnancy exerts a more significant impact. Nutrients and dietary compounds consumed by the mother not only support fetal growth but also contribute to shaping the initial fetal microbiome, which in turn impacts neurodevelopment (118, 119). Early pregnancy and infancy are critical phases for brain development, setting the stage for the formation of cognitive, motor, and socio-emotional functions that will continue to evolve throughout childhood and adulthood (120). Specifically, the prenatal period around 22 days after conception is considered a critical window for neural tube formation. At this stage, sufficient nutrient intake, such as folic acid, copper, and vitamin A, is essential for neural tube closure and prevents birth defects in the brain and skull (120). During this critical period, fetuses and infants are susceptible to environmental factors, especially nutritional deficiency. Therefore, sufficient nutrient supply is critical to support the rapid trajectory of key neurodevelopmental milestones, including synapse formation and myelination (121). Moreover, the postnatal periods from birth to early childhood are considered a sensitive period and “window of opportunity” where the brain continues to develop but retains some plasticity, allowing it to respond to environmental stimuli and nutrition intervention (122). The impacts of nutritional deficiencies on neurogenesis and synaptogenesis during prenatal development are often irreversible, as these critical developmental events are tightly programmed during specific phases of embryogenesis. Studies indicate that an unbalanced maternal diet or key nutrient deficiency can result in neurobehavioral delays, potentially affecting cognitive and behavioral outcomes in children (123). In contrast, nutrient deficiencies during the postnatal period may be recoverable due to the brain’s neural plasticity, allowing for functional adaptation and recovery at this stage (124).

Both macro-and micronutrients are essential for neural development. Malnutrition-induced energy deficits or excesses will not only result in stunting growth but also lead to impaired cognitive function among children (125). Any deficiency of these nutrients during the critical and sensitive window of development may cause long-term impacts on brain structure and functions (126), as well as organizational events such as neurogenesis, cell migration, differentiation, and glial cell function (127). Among the key nutrients, polyunsaturated fatty acids (PUFAs), particularly omega-3 fatty acids like docosahexaenoic acid (DHA) and eicosapentaenoic acid (EPA), are crucial for brain development. DHA is a key structural component of neuronal cell membranes. DHA level changes can influence cell membrane-associated proteins and the function of protein receptors (128). Unlike DHA, EPA acts as a precursor for eicosanoids and a modulator of cytokines, which play roles in neurotransmission and neuromodulation (128). Brain plasticity and learning ability in mice can be impacted by omega-3 PUFA supplementation due to its effects on enhancing hippocampal levels of neurotrophic factors, reducing oxidative stress, and metabolic effects (129). In human studies, the effectiveness of omega-3 PUFA supplementation in psychiatric disorders has been reported, including ADHD, ASD, and depression, although the results remain controversial (130, 131).

Vitamin A, a fat-soluble micronutrient, can be converted to its bioactive form, retinoic acid (RA), and acts as a transcriptional regulator. RA is essential for CNS development through the regulation of neurodifferentiation and neuronal patterning. RA also maintains brain plasticity and neural stem cell production in the mature CNS. Therefore, the disrupted RA signaling may lead to motor neuron diseases and degenerative diseases (132). In mice, vitamin A deficiency not only led to physical and ocular abnormalities but also impaired long-term potentiation and depression, along with declined cognitive skills (133). Vitamin D is essential not only for bone health but also for neurodevelopment as a fat-soluble vitamin. It regulates the biosynthesis of neurotransmitters and neurotrophic factors. The abundance of vitamin D receptors in the fetal brain highlights its crucial role in early neurodevelopment (134). In mouse studies, vitamin D deficiency in mothers or the offspring impaired learning abilities and memory and increased the risk of psychiatric disorders later in life (135). Similarly, human studies found that prenatal vitamin A and D sufficiency decreased the risk of schizophrenia and other mental disorders later in life (136). Preclinical studies have explored the role of maternal folate in neurodevelopment and brain functioning in the offspring, including DNA replication, gene expression, and synthesis of phospholipids and neurotransmitters (137). Human studies further support a positive association between maternal folate intake during pregnancy and cognitive function in the offspring (138). When it comes to minerals, zinc and copper are the trace elements that are vital for neuronal functions. Zinc is essential for normal neurogenesis, neuronal migration, myelination, synaptogenesis, and regulation of neurotransmitters. Insufficient zinc intake in the postnatal period can have significant impacts on mental and cognitive development (139). Copper deficiency may lead to abnormal brain development, as evidenced by Menkes disease, a neurodegenerative disorder in infants and young children due to defective copper transport (140). The impaired metabolism and signaling of zinc and copper have been reported in brain diseases and psychiatric disorders, including ASD and major depressive disorder (141, 142). In summary, prenatal and early-life nutrition play a fundamental role in shaping brain development. Deficiencies and imbalances in these nutrients may lead to adverse neurological consequences in the long run.

## 5 Impacts of dietary patterns on the gut microbiome and brain development

Although many studies focus on analyzing the impacts of individual nutrients, assessing diet intake as a whole is essential for exploring the interplay between food components and nutrients that contribute to infant neurodevelopment and behavior (143). Maternal malnutrition, whether in the form of undernutrition or overnutrition, can have profound impacts on early fetal neurodevelopment and increase the risk of various neuropsychiatric conditions later in life (123). Poor maternal diet quality has been associated with lower intelligence scores (9, 144, 145), emotional and behavioral dysregulation (143, 146), and hyperactivity-inattention symptoms (147, 148). In contrast, a healthier maternal dietary pattern characterized by a higher intake of fruits, vegetables, and fish while limiting red meat and trans fats has been linked to fewer behavioral difficulties and improved executive function in the offspring (143). This section will discuss several common dietary patterns and their impact on brain development and the gut microbiome in offspring, as shown in Table 1.

**Table 1:** Correlation of dietary patterns with neurodevelopmental outcomes and the gut microbiome.

Dietary patterns	Animal models	Human studies	Gut microbiome
High-fat diet	Maternal or early-life HFD disrupts hippocampal neurogenesis, increases brain lipid peroxidation and inflammation, alters brain structure, impairs spatial learning and upregulates ASD-related genes (152, 169) HFD exposure elevates microglial activation and proinflammatory cytokines in the offspring hippocampus, leading to behavioral deficits (175–177)	Maternal prepregnancy obesity and diabetes are related to an increased risk for ASD and intellectual disabilities in the offspring (153–155, 161, 162) Maternal pre-pregnancy BMI increment in neurocognitive function in children may contribute to an increased risk of ADHD in children (22, 156–158, 163, 164) Maternal pre-pregnancy BMI increment may influence the development of internalizing and externalizing symptoms, leading to a high risk of emotion dysregulation and intensity in the offspring (159, 165–167) Maternal obesity is linked to poorer visual-motor abilities and verbal recognition due to maternal inflammation (160, 168)	Maternal HFD disrupts the gut microbiome, impairing gut-brain signaling and synaptic plasticity, contributing to neurodevelopmental and psychiatric disorders in the offspring (178–180) HFD increases microbial gene activity in the maternal gut, which impacts neuronal plasticity and glutamate metabolism (178) Maternal HFD causes gut dysbiosis with reduced *L. reuteri*, which is essential for social bonding and emotional regulation (181) Maternal HFD reduced maternal SCFA butyrate levels due to lower levels of butyrate-producing bacteria (*Lachnospira*, *Ruminococcus*) (184)
Ketogenic diet	Gestational KD may support early brain development, but excessive ketone exposure can cause neonatal brain abnormalities and risk of ketoacidosis (189) KD treatment changes brain structures and shows improvements in ASD deficits related to myelin formation and white matter development (191, 195) ASD symptoms were improved by KD in animal models of different etiologies (196–199) KD improves ADHD-related behavioral factors in canine models, like chasing, excitability, and trainability (204)	KD improves ASD symptoms, enhances cognition and sociability, and ameliorates comorbidities such as ADHD and sleeping disorders (200–203) KD improves behavioral and cognitive functioning in children and adolescents, especially those with seizure disorders (194–197) KD can potentially worsen the emotional problems in children (198)	KD alters the *Firmicutes*-to-*Bacteroidetes* ratio by increasing *Clostridium cluster XI* and *Bacteroidetes*, and reducing *Firmicutes* in ASD patients (215, 216) KD increases SCFA-producing bacteria such as *Akkermansia muciniphila* and *Lactobacillus* while decreasing harmful *Desulfovibrio*, supporting BBB integrity and neurovascular development in the offspring (213) KD increases gut microbiota diversity, raising abundances of *Ruminococcus_gauvreauii_group*, *Bacteroides*, and *Bifidobacterium*, linking to reduced GABA production, which is deficient in ADHD (205)
Mediterranean diet	In the mouse model of chronic inflammation, MD improves recognition and memory along with the shifts in the brain proteomic profile (221) MD improves the gut and brain inflammatory profiles, aligning with enhanced neurocognitive and neuromuscular functions (246)	MD has been shown to improve children’s IQ development and overall neurodevelopment (144, 225, 227) Low adherence to MD may lead to higher susceptibility to externalizing problems and anxiety, depression, ADHD, and autistic traits (219, 228, 229) MD supports healthy brain development and structure like total fetal brain volume, corpus callosum, and right frontal lobe (230, 231).	MD shifts the gut microbiome composition, restores the *Firmicutes*-to-*Bacteroidota* ratio, and increases bacterial species such as *Muribaculum*, *Rikenellaceae Alloprevotella*, and especially *Akkermansia* (221) MD supports the growth of *Lactobacillus*, *Akkermansia*, *g_Erysipelatoclostridiaceae*, *Lachnoclostridium*, *Intestimonas*, and *Parasutterella*, which are known for producing lactate, enhancing neurocognitive and neuromotor functions (246, 247)
Plant-based diet	Relevant animal studies are not found.	PD adherence is associated with increased productivity and improvements in anxiety, stress, and depressive symptom scores (250, 251, 254, 255) Low meat intake was related to depressive symptoms, and no protective effects were found (252, 253) Children in the highest quartile of the PD Index have lower odds of ADHD symptoms (256, 257)	A vegan or vegetarian diet decreases SCFA levels (263) PD can lead to increased levels of *Prevotella* and decreased *Bacteroides*, contributing to altered metabolic pathways involved in neurotransmitter production (258) PD increases the butyrate-producing bacteria, such as *Ruminococcacea*e, *Lachnospiraceae*, *Coprococcus*, *Roseburia*, *Blautia*, *Alistipes,* and *Faecalibacterium prausnitzii*, which are crucial for neuron function and maturation (264)

### 5.1 High-fat diet

In the United States, a significant proportion of women of childbearing age face weight-related health challenges. Nearly 60% of women aged 20 to 39 are classified as overweight, with one-third falling into the category of obesity. Additionally, around 16% of women in this age group are diagnosed with metabolic syndrome (149). Maternal obesity and overweight can lead to gestational diabetes and other metabolic disorders that may affect fetal development (150). Maternal metabolic disruptions, including insulin resistance, elevated blood glucose, and systemic inflammation, can interfere with critical stages of fetal brain development (151). Energy-dense diets such as high-fat diets (HFD) or Western-type diets are primary dietary patterns that contribute to overweight and obesity as well as related metabolic disorders.

Maternal HFD can directly affect the intrauterine environment, which, in turn, has significant implications for infant brain development and behavior. Maternal HFD can induce increased brain lipid peroxidation, altered hippocampal neurogenesis, and heightened inflammation, which negatively affect neurodevelopmental processes, leading to an increased risk of emotional and behavioral disorders in the offspring (152). Maternal obesity-associated neurodevelopmental conditions such as ASD (153–155), ADHD (156–158), depression, anxiety (159), and cognitive functions (160) are the most common outcomes observed in cohort studies (151). Specifically, pre-pregnancy maternal obesity and diabetes have been linked to a higher risk of ASD and intellectual disabilities in the offspring. The risk increases significantly when obesity and diabetes co-occur (153). Recent studies revealed that maternal BMI is considered a potent biomarker for offspring ASD risk (161, 162). A large cohort study found that each unit increase in pre-pregnancy BMI was linked to a 3% higher odds of children having a high ADHD symptom score (22), consistent with findings from many other studies (163, 164). Few epidemiological studies focus on the association between maternal HFD and the risk of depression and anxiety in the offspring. Studies found that maternal BMI is related to a two-fold increased risk of emotion dysregulation and intensity in the offspring (163, 165). Interestingly, either being small or large for gestational age also increases the odds of depression later in life (166, 167). Besides, maternal dietary patterns contribute to cognitive functions in the offspring, with high maternal BMI specifically linked to poorer visual-motor abilities and verbal recognition (160, 168). Maternal HFD exposure has a long-term impact on brain development, resulting in brain structural changes, which can persist into adulthood. Studies found that both regional and total brain volumes were significantly affected by early-life HFD exposure, with a positive correlation between dietary fat percentage (169). Additionally, they identified several genes that are highly expressed in brain regions sensitive to early-life HFD exposure, which are related to feeding behavior and autism in both mice and humans (169).

As shown in Table 1, animal studies and human clinical trials have explored potential mechanisms linking maternal HFD intake to offspring neurodevelopmental conditions. It is believed that maternal HFD consumption impairs offspring neurodevelopment by triggering inflammation in the maternal gut, adipose tissue, and placenta, leading to elevated proinflammatory cytokine levels in both the mother and fetus (151). These increased proinflammatory cytokines include various interleukins, tumor necrosis factor-alpha (TNF-*α*), and interferon-gamma (IFN-*γ*), creating a state of chronic low-grade inflammation in the mother, which can negatively impact the neurodevelopment of the offspring (151, 170). For example, elevated levels of IL-6 in mothers have been associated with reduced social behaviors in the offspring (171). In contrast, elevated IL-17a has been shown to contribute to abnormalities in brain development, particularly in the cortex (172). Maternal hyperglycemia, induced by HFD, triggers excessive proinflammatory cytokine production in the placenta. Neonatal hyperglycemia also increases IL-1β, TNF-*α*, and toll-like receptor activity in spleen cells, causing chronic low-grade inflammation in infants. All these changes activate microglia, induce the inflammatory response in the CNS, and impair fetal and neonatal brain development (173). As a result, these proinflammatory cytokines are linked to a higher likelihood of alterations in cognition and behavioral disorders (174, 175). For example, studies in rodent models showed increased microglial activation markers and proinflammatory cytokine levels in the hippocampus of offspring exposed to maternal HFD (176). Some evidence suggests that brain development during lactation is also sensitive to HFD. Maternal HFD during lactation can cause inflammation in the offspring’s brain, leading to impaired axon formation, particularly in the hypothalamus, and reduced arcuate nucleus neuronal fiber densities (175, 177).

Recent studies reveal a potential causal link between maternal HFD-induced gut microbiome dysbiosis and offspring neurodevelopmental conditions. Maternal HFD can cause a disrupted gut microbiome, leading to gut-brain signaling dysregulation that impacts synaptic plasticity, which is essential for proper neuronal communication and development. Synaptic plasticity is considered an early site where mental disorders may begin to develop (178). Impaired synaptic function, driven by these microbiome changes, can negatively affect social behaviors in the offspring and has been linked to the onset of neurodevelopmental and psychiatric disorders (179, 180).

A growing amount of evidence implicates the potential role of the gut microbiome in neurodevelopment and behaviors. Animal studies found that microbial community alterations induced by maternal HFD consumption influence the offspring’s synaptic function and behavior (178). Specifically, they found that maternal HFD increased microbial gene activity in the maternal gut, leading to changes in quinolinic acid production and kynurenine levels, which are important for neuronal plasticity and glutamate metabolism. During adolescence, offspring exposed to maternal HFD showed heightened locomotor activity and anxiety-like behaviors, along with an increase in glutamate-related gene expression (178). Similarly, another study reported that maternal HFD caused gut dysbiosis in the offspring. A specific gut bacterial species, *Lactobacillus reuteri (L. reuteri),* was significantly reduced in the offspring exposed to maternal HFD. Interestingly, reintroducing *L. reuteri* significantly improved social interactions (181). *L. reuteri* has been shown to promote the production of oxytocin, a hormone involved in social bonding, trust, and emotional regulation (182). Oxytocin plays a crucial role in maternal care, pair bonding, and social recognition, making it essential for healthy social interactions (183). Moreover, beneficial microbial metabolites such as SCFAs are crucial for normal fetal brain development. In animal studies, an HFD resulted in a decreased level of butyrate-producing bacteria, like *Lachnospira* and *Ruminococcus*, contributing to lower butyrate levels in HFD-exposed mothers (184). HFD can regulate SCFA receptor expression in adipose tissue. These receptors are also found in fetal and uteroplacental tissues, suggesting their roles in maternal gut-fetal brain signaling (151). To conclude, maternal HFD and obesity can negatively impact offspring neurodevelopment by inducing neuroinflammation, oxidative stress, and altered synaptic plasticity, which may be due to HFD-induced gut microbiome dysbiosis.

### 5.2 Ketogenic diet

The ketogenic diet (KD) is a nutritional approach emphasizing high fat and low carbohydrate intake. KD typically contains approximately 60% fat, 30% protein, and 10% carbohydrate. By drastically reducing around 50% of carbohydrates, the body enters a metabolic state called ketosis, primarily burning fat for energy instead of glucose (185). The KD was initially used for treating pediatric epilepsy and has now become a tailored dietary regimen facilitating weight loss in obese and overweight patients (185). However, its effects on fetal neurodevelopment remain largely unknown. By following KD for a few days, several organs, including the brain, experience a shift from glycolysis to ketosis. Notably, fat is a primary component in breast milk, suggesting a neonatal preference for ketone bodies as a key energy source in early development (186, 187). Besides, astrocytes, oligodendrocytes, and neurons from the developing brain can use ketone bodies for respiration (186). Ketone bodies, a group of liver-produced fat metabolites, help to prevent the mitochondrial permeability transition and reduce the generation of reactive oxygen species (188). The functions of ketone bodies in CNS cell development have been reported (187). Studies have shown increased enzymes for ketone utilization during gestation that support early brain development, specifically lipid and white matter synthesis (189). However, excessive ketone bodies may have adverse effects on fetal brain development because of the impaired production of nucleic acids, which are crucial for cell growth and development (189). KD broadly impacts brain function, influencing multiple cellular and molecular processes. Specifically, KD can alter gene expression and modify neurotransmission. Evidence has shown that KD shifted the balance between excitatory and inhibitory signaling, which can stabilize neural networks (190). In an animal study, KD treatment showed improvements in myelin formation and white matter development, possibly attributed to the altered neurotransmitter signaling pathways (191). Additionally, KD increases levels of neurotrophic factors to support neuron growth and resilience. Protein phosphorylation is also affected by KD, leading to modified key signaling pathways involved in memory and learning (192). KD also alters amino acid metabolism, potentially enhancing the production of neurotransmitters like GABA (193). KD has been found to inhibit the mammalian target of the rapamycin (mTOR) signaling pathway, leading to anticonvulsant effects. The mTOR plays a key role in cell growth, metabolism, and autophagy, which are associated with the progression of ASD (194).

Prenatal and early postnatal KD treatment in mice led to structural alterations in the offspring’s brains (189, 195). The cortical volume showed slight bilateral enlargement, while the midbrain volume was reduced unilaterally. During the postnatal period, cortical enlargement became more pronounced, and the hypothalamus was relatively larger, whereas the hippocampus, corpus callosum, and olfactory bulb were relatively smaller (189). These alterations in brain structure may correspond to functional and behavioral changes in KD-exposed offspring, though further postnatal behavioral studies are needed to clarify these effects. Although the clinical evidence is limited due to the difficulties of implementing the KD diet in humans, maternal KD exposure has been shown to influence offspring behavior due to alterations in brain structure and neurodevelopmental processes in mice (189).

Nevertheless, KD has been shown to improve ASD core symptoms and comorbidities like seizures in mice (196). Prenatal KD reversed the abnormalities of maternal immune activation-induced ASD, while control diet-fed mice demonstrated repetitive self-directed behaviors (196). However, such an effect was only observed in male offspring, indicating potential sex-dependent differences in response to KD (196). This finding is consistent with other animal models focused on different etiologies of KD (197–199). For example, animals with ASD induced by prenatal valproic acid showed improved social behaviors after KD treatment (199). Another study found that KD enhanced brain activity related to social novelty in Engrailed 2 gene knockout mice, as indicated by increased levels of neuronal activation in specific brain regions (198). In a human study, one patient out of six showed significant improvement on the Childhood Autism Rating Scale, and the other five showed milder symptom improvements after KD treatment. Overall, KD ameliorates comorbidities such as ADHD and sleeping disorders in ASD patients (200). Other studies showed similar results, suggesting significant improvements in cognition and sociability in children with ASD who underwent a KD treatment (201–203). However, a larger scale of clinical studies is necessary to validate the effectiveness of KD treatment in pediatric patients with behavioral conditions.

Evidence on KD treatment in ADHD patients is scarce. One study using a canine model with naturally occurring epilepsy and comorbid ADHD showed that KD improved ADHD-related behaviors such as chasing, excitability, and trainability (204). In a spontaneously hypertensive rat (SHR) model, KD treatment ameliorated the relevant symptoms, similar to methylphenidate, the first-line medication for ADHD patients. Importantly, this study found that KD increased the abundance of *Bacteroidota* in SHR rats (205). Besides, KD has been shown to exert antidepressant and mood-stabilizing effects on managing both physical and mental symptoms, mainly due to its impact on regulating GABA and glutamate levels, which are critical for maintaining brain functions and dopamine levels (206). In addition, the shifts in brain energy metabolism induced by KD may help counteract the global cerebral hypometabolism often observed in depression and bipolar disorder. Another potential benefit of ketosis is its ability to lower intracellular sodium levels, possibly contributing to mood stabilization (207). One randomized controlled clinical trial showed that children with epilepsy on KD enhanced their behavioral and cognitive functioning, demonstrated by lower anxiety and hostility levels, higher productivity, and improved attention (208), which is consistent with other observational studies (208–211). However, the effectiveness of KD remains controversial. For example, some studies revealed a tendency of worsening emotional problems among children on KD (212).

The gut microbiome plays a vital role in mediating the therapeutic effects of KD on managing CNS disorders. An increasing amount of research supports the potent therapeutic effects of KD on neurological disorders or illnesses via remodeling the gut microbiome (213). One study used a rodent model of ASD to validate the gut microbiome disturbances in the case of ASD compared to the control group. The results showed that the total amount of bacteria was significantly reduced because of the antimicrobial-like effect of KD (214). Specifically, the gut microbiome revealed notable shifts in mice that resembled similar changes observed in humans with ASD (215). These changes included an increase in *Clostridium cluster XI* and *Bacteroidetes* and a decrease in *Firmicutes* species (215, 216). KD increased the *Firmicutes*-to-*Bacteroidetes* ratio, counteracting the low ratio commonly seen in ASD phenotypes. Meanwhile, KD increased the SCFA-producing bacteria, including *Akkermansia muciniphila* and *Lactobacillus*, twofold to threefold in mice following KD (215). KD intake also decreased the levels of *Desulfovibrio*, a harmful bacterium. Taken together, these changes in the gut microbiome support the potential protection of KD in maintaining the integrity of the BBB, which further promotes neurovascular development (213). Generally, the beneficial effects of KD are mainly attributed to its impacts on the gut microbiome by decreasing the *α*-diversity and species richness (213). However, this remains controversial. For example, the study in SHR rats found that KD increased the richness and diversity of the gut microbiota at the phylum, family, and genus levels (205). Additionally, KD treatment can alleviate ADHD symptoms in SHR by enhancing gut microbiota-driven amino acid and glucose metabolism (205). While KD has shown promising effects in improving ASD, ADHD, and mood disorders by stabilizing neural networks and reducing neuroinflammation, its long-term effects on fetal brain development remain unclear. More clinical studies are needed to determine the efficacy of maternal and neonatal KD in pediatric neurodevelopmental disorders.

### 5.3 Mediterranean diet

Mediterranean diet (MD) is a well-known healthy dietary pattern rich in unprocessed food, vegetables, fruit, whole grains, nuts, legumes, fish, and olive oil, while sparse in red meat. MD can provide potent health benefits against various human chronic diseases, such as cardiovascular diseases, diabetes, metabolic-related conditions, and cancers (217). MD stands out for its high amount of monounsaturated fatty acids, omega-3 and omega-6 PUFAs, and antioxidants, contributing to healthy neurological and behavioral development in children (218, 219). MD consumption has been related to the methylation changes in inflammation-related genes (220). In a mouse model with chronic inflammation, MD improved recognition memory, the shifts in the gut microbiome composition, and the brain proteomic profile (221). In human studies, MD consumption supplemented with olive oil and nuts improved cognition in the elderly (222).

Many studies have shown that MD adherence can lead to improved pregnancy and birth outcomes, including a reduced risk of gestational diabetes, hypertension, preterm delivery, and intrauterine fetal growth restriction (223). Recent research focused on the impacts of maternal MD on behavioral development showed that low intake of fish and high intake of processed food were related to early-onset of persistent conduct problems in children (224). A diet high in vegetables and fruits and low in meats, similar to the MD, has been shown to enhance children’s IQ development (144). Similar results support the beneficial effects of maternal MD adherence on improving the offspring’s brain development and reducing the risk of neurodevelopmental and behavioral disorders (147, 225–227). The low adherence to maternal MD may lead to higher susceptibility to anxiety, depression, ADHD, and autistic traits in children (219, 228). In contrast to MD, an unhealthy dietary pattern characterized by a high intake of processed foods and sweets has been related to an increased risk of internalizing and externalizing problems (229) as well as hyperactivity-inattention symptoms in children aged between 5 and 8 years old (147). As mentioned before, unhealthy diets such as HFD or a Western diet can cause unbalanced growth factors and neurotransmitters, leading to abnormal behavioral development in children. On the contrary, the main components of MD, such as antioxidants and beneficial fatty acids, support healthy brain development (230). Additionally, studies focused on fetal outcomes and found that those offspring exposed to maternal MD had larger total fetal brain volume, corpus callosum, and right frontal lobe, contributing to optimized brain structure and neurological functions (231). Nevertheless, direct evidence connecting MD consumption to behavioral outcomes in the offspring remains largely unexplored.

Studies have examined the impacts of MD consumption during the postnatal period. The results showed the beneficial effects of MD on improving cognitive function and school performance (232), lowering the risk of depression (233) and ADHD (234), along with metabolic protective effects on BMI, glucose, and lipid profiles in children (235). Therefore, MD has been recognized as a promising strategy for treating depression and alleviating systemic inflammation among children and adolescents (236). MD was first tested and used as an adjuvant treatment strategy among young adults with major depressive disorder in the “SMILES” trial (237). One study found that the MIND diet, a combination of MD and Dietary Approaches to Stop Hypertension (DASH) diet for Neurodegenerative Delay, was effective in improving depressive symptoms, slowing cognitive decline in the elderly, and reducing the risk of anxiety disorder in adults (238, 239). Another case–control study in Iranian children found that high adherence to the MIND diet was closely related to lower odds of ADHD (240). More longitudinal studies are required to explore the impacts of the MIND diet on brain health in children and adolescents.

While some evidence suggests the potential effects of a maternal MD on offspring neurodevelopment, findings on the specific mechanisms remain limited. MD is abundant in essential vitamins, minerals, flavonoids, polyphenols, and other phytochemicals that help reduce oxidative stress and inflammation in neural tissues (241). Additionally, epigenetic modifications may provide a possible explanation for the observed association. Studies have found that the hypomethylation at the maternally expressed 3 imprinting gene relates to adverse neurobehavioral phenotypes (230, 242). In the context of mental and behavioral problems, significant methylation changes have been observed in schizophrenia and bipolar disorder (243) and ADHD symptoms in children (244). Intriguingly, maternal MD adherence has been found to be associated with improved behavioral outcomes in the offspring, accompanied by DNA methylation changes at specific imprinted differentially methylated regions (245). More studies are needed to support the early intervention of MD for preventing or treating neurodevelopmental disorders in the offspring.

MD consumption significantly affects the gut microbiome ecosystem. Research found that MD supported the growth of beneficial bacteria such as *Lactobacillus*, *Akkermansia*, *g_Erysipelatoclostridiaceae*, *Lachnoclostridium*, *Intestimonas*, and *Parasutterella*, known for SCFA-producers that play a key role in enhancing neurocognitive and neuromotor functions (246, 247). These bacterial compositional alterations also contribute to inhibiting systemic inflammation and oxidative stress. Supportively, MD-fed mice displayed more KEGG pathways, particularly those involved in D-alanine metabolism, glycosaminoglycan breakdown, steroid hormone biosynthesis, and pathogen defense (246). MD improved gut and brain inflammatory profiles, such as reduced levels of cytokines including IL-1β, IL-6, and TNF-*α*, leading to enhanced neurocognitive and neuromuscular functions (246). Behavioral improvements by MD treatment were accompanied by shifts in microbiota composition, including the restored *Firmicutes*-to-*Bacteroidota* ratio and increases in *Muribaculum*, *Rikenellaceae Alloprevotella*, and especially *Akkermansia*, which strongly correlated with improved performance (221). Moreover, research indicates that maternal adherence to the MD influenced the fecal gut microbiome with an increased abundance of bacterial species such as *Ruminococcaceae*, *Acidaminococcaceae*, and *Bacteroidaceae* (248). Although many studies are exploring the effects of MD on children’s neurodevelopment, mechanistic exploration focused on the gut microbiome correlation is still insufficient. More studies in this area are needed to further validate the causal link between MD consumption and improved neurodevelopmental and behavioral outcomes in children and the gut microbiome changes.

### 5.4 Plant-based diet

A plant-based diet (PD) emphasizes the consumption of food derived from plants with a limited intake of animal products. Due to the rich content of antioxidants and anti-inflammatory components, as well as its low calorie content, PD is also effective in reducing age-related cognitive decline (249). Intervention trials in healthy adults found that PD adherence was associated with increased productivity and improvements in anxiety, stress, and depressive symptom scores (250, 251). However, some controversial results showed that low meat intake was related to depressive symptoms or no protective effects were found (252, 253), while others showed protective effects associated with PD intake (254, 255). One case–control study found that children in the highest quartile of the PD Index had higher energy, macronutrient, and key micronutrient intake with significantly lower odds of ADHD than the counterparty (256). Similarly, another study on preschoolers in China found that “vegetarian” or PD dietary patterns were negatively correlated with ADHD symptoms (257).

Overall, research on PD impacts on neurological and psychiatric disorders is limited as compared to MD, which has been well studied for its neuroprotective benefits (258). The potential mechanism linking PD to brain health may involve reduced systemic inflammation (259). This can be attributed to the high intake of anti-inflammatory plant compounds, such as polyphenols, flavonoids, and fiber, as well as the avoidance of pro-inflammatory molecules in animal-derived foods. While PD offers numerous health benefits, it is possible that exclusive PD can cause nutritional deficiencies, contributing to increased depressive symptoms (254). Key nutrients such as vitamin B12, iron, omega-3 fatty acids, and zinc are rich in animal-based foods and play crucial roles in neurotransmitter function, brain structure, and mood regulation (260). Therefore, infants and children, as well as their mothers, following a strict vegetarian or vegan diet during pregnancy, may be at a higher risk of impaired neurodevelopment. Nevertheless, there is little direct evidence to show the negative impacts of a maternal vegetarian or vegan diet on children’s cognitive function (261).

The microbiome composition affects the availability of neurotransmitter precursors, such as tyrosine and tryptophan, which are essential for the synthesis of dopamine and serotonin (262). The levels of SCFAs are closely linked to the intake of fruits, vegetables, and legumes. However, a strict vegan or vegetarian diet decreases SCFA levels, potentially due to weight loss and a high intake of low-fermentable non-starch polysaccharides (263). Notably, PD can lead to increased levels of *Prevotella* and decreased levels of *Bacteroides*, altering metabolic pathways involved in neurotransmitter production (258). Also, studies have reported that PD led to an increased abundance of butyrate-producing bacteria, such as *Ruminococcaceae*, *Lachnospiraceae*, *Coprococcus*, *Roseburia*, *Blautia*, *Alistipes,* and *Faecalibacterium prausnitzii* (264). Lower inflammation-associated PD may contribute to improved cognitive function and mental well-being (265). In summary, while PD may support brain health through anti-inflammatory and microbiome-related mechanisms, its long-term neurological effects on neurodevelopment and mental health in children remain inconclusive and require further investigation.

## 6 Impacts of bioactive nutrients on neurodevelopment and the gut-brain axis

### 6.1 Broccoli sulforaphane

Sulforaphane (SFN), a bioactive compound abundant in cruciferous vegetables such as broccoli, Brussels sprouts, and kale, has gained significant attention for its neuroprotective and chemopreventive properties (266, 267). SFN can act as an inducer of the nuclear factor erythroid 2-related factor 2 (Nrf2), regulating the antioxidant response element pathway and delivering protective effects against various neurodevelopmental diseases and behavioral disorders (266).

As shown in Table 2, preclinical research on the neuroprotective effects of SFN has yielded promising results over the past few decades. SFN plays a crucial role in supporting mitochondrial and synaptic function, which leads to reduced neuroinflammation and promotes neuroprotection. SFN exerts its bioactive effects by regulating key genes involved in cellular defense and damage repair, thereby enabling cellular activities that counteract oxidative stress, inflammation, DNA-damaging agents, and radiation (268). The anti-inflammatory properties of SFN can further mitigate cytotoxicity in the nervous system, thereby exerting neuroprotective effects (269). In a placebo-controlled, double-blind, randomized trial, a group of participants aged 13–27 with moderate to severe ASD who received SFN treatment demonstrated improvements in social interaction, abnormal behavior, and verbal communication, partially due to SFN-included protective effects on oxidative stress, mitochondrial function, lipid peroxidation, and neuroinflammation (268). These results are consistent with the outcomes of other clinical trials that focused on SNF-led neuroprotective effects in children and young adults (270–274). However, the significance of the protective effects remains uncertain (275).

**Table 2:** Bioactive compounds on neurodevelopment and gut bacteria.

Bioactive compounds	Animal models	Human studies	Gut microbiome
Broccoli Sulforaphane	SFN alleviated depression-like behaviors by reducing TNF-α levels, microglial activation, and inflammation. It also enhanced neuroprotection and plasticity (276–278) SFN alleviated the ASD symptoms in mice and humans, evidenced by the improved verbal and non-verbal communication score, possibly due to the activation of Nrf2 (282, 283)	ASD patients with SFN treatment improved social interaction, abnormal behavior, and verbal communication (268, 270–274) Maternal supplementation of SFN may prevent behavioral abnormalities in the offspring via the MIA, a known factor in the development of neuropsychiatric disorders (284) SFN supplementation during adolescence prevented the phencyclidine-induced cognitive deficits in schizophrenia during adulthood (285)	SFN increased *Lactobacillus, Bifidobacterium, Ruminococcaceae UCG-014, and Intestinibacter*, and genera like *Oscillibacter, Enterorhabdus, Blautia,* and *Lachnospiraceae* were less abundant (286) SFN increased the beneficial bacteria such as *Bifidobacterium* and *Lactobacillales* and decreased *Parasutterella* in ASD children (282)
Tea polyphenol	In a sleep-deprived mouse model, TPs improved recognition ability and reduced anxiety-like behaviors by suppressing TNF-α production (298) TPs improved locomotor activity and spatial cognition learning ability in mice (299, 300) Catechin ameliorated behavioral, biochemical, neurological, and molecular impairments in ASD models via the nitric oxide pathway (302) Prenatal green tea consumption led to increased locomotor activity, decreased anxiety, and improved memory and learning abilities in the offspring (288)	In young adults, long-term tea consumption promoted reward learning and prevented depression-related symptoms (303) In a cross-sectional study, higher consumption of green tea was associated with a lower prevalence of depression (304)	Catechins increased *Bifidobacterium* and *Lactobacillus/Enterococcus* groups and reduced the abundance of *Bacteroides*-*Prevotella*, *Clostridium histolyticum*, and *Eubacterium-Clostridium* groups, along with the increased concentration of SCFAs (306, 307) TPs can inhibit harmful bacteria like *H. pylori, S. aureus, Bacteroides,* and *L. monocytogenes* while promoting beneficial probiotics such as *Bifidobacterium* and *Lactobacillus-Enterococcus* (309)
Soy Isoflavone	High level of prenatal soy diet related to increased risk of offensive and depressed behaviors in male mice (323, 326) Maternal exposure to soy isoflavone, daidzein, during pregnancy and lactation resulted in enhanced social affiliation and improved spatial memory in female offspring (327)	A moderate level of prenatal isoflavone exposure relates to a decreased risk of childhood depression and anxiety. The highest level of prenatal exposure negatively impacted behavioral health (324) High prenatal isoflavone consumption reduced the risk of hyperactivity in children (325) Soy formula during infancy was related to reduced female-typical play behavior (328) A potential association between soy formula and autistic behaviors was found (329).	Soy isoflavone treatment enhanced intestinal homeostasis by reducing the *Klebsiella*, *Pseudomonas*, *Acinetobacter*, *Escherichia-Shigella*, and *Staphylococcus*. The beneficial bacteria, including *Bifidobacterium*, *Bacteroides*, *Dorea*, *Faecalibacterium*, and *Lactobacillus*, significantly increased (332) Soy isoflavones decreased *Firmicutes* species and lowered the *Firmicutes*-to-*Bacteroidetes* ratio while increasing *Proteobacteria* and *Actinobacteria* compositions (333)
Curcumin	Curcumin exerted antidepressant effects by inhibiting the immobility period, increasing serotonin and dopamine levels, as well as inhibiting the MAO enzymes (338) Curcumin improved memory and learning abilities by boosting the cholinergic system and antioxidant activity (339, 340) Curcumin alleviated toxins-induced neurodevelopmental deficits, restoring neurotransmitter balance and reducing anxiety in the offspring (345–350) Curcumin restored key neurotransmitters, enhanced α7-nicotinic acetylcholine receptors, reduced oxidative stress, and alleviates ASD-like behaviors (326, 348, 351) Mice that followed curcumin treatment exhibited less anxious and hyperactive behavior in the open field test and the social interaction test (352)	Curcumin improved the symptoms of major depressive disorder, along with improved depression-related biomarkers (353–355)	Curcumin improved gut microbial balance by increasing beneficial *Bifidobacteria* and *Lactobacilli* while reducing pathogenic *Prevotellaceae*, *Coriobacterales*, *Enterobacteria*, and *Enterococci* (359) Curcumin reduced *Prevotellaceae, Bacteroidaceae, and Rikenellaceae*, which are likely related to systemic diseases (357, 360) Curcumin and its metabolite tetrahydrocurcumin enhanced antioxidant enzyme activity and modulated PI3K/Akt, AMPK, and Nrf2 pathways to support neuronal survival (357, 361)

In animal models, SFN has been shown to attenuate lipopolysaccharide-induced depression-like behaviors by inhibiting TNF-*α* in the serum and suppressing microglial activation in brain regions (276). Consistently, in another mouse model, SFN provided neuroprotective and antidepressant effects by regulating glucocorticoid receptor expression and reducing inflammation-induced apoptosis and disrupted neuroplasticity, potentially through the NF-κB and ERK/CREB/BDNF signaling pathways (277, 278). Another study confirmed the effects of repeated SFN treatments in reversing depression-like behaviors and increasing the total antioxidant capacity in animal models (279). As a lower Nrf2 level is correlated with depression, SFN may induce antidepressant effects by inducing Nrf2 activation (280). A clinical study investigated the effects of SFN supplements in depressed patients and showed improvements in depressive symptoms caused by cardiac interventions (281). In addition to its antidepressant effects, SFN has been found to alleviate symptoms of ASD in mice and humans, as supported by improved verbal and non-verbal communication scores (282). In BTBR mice, SFN reduced Th17 immune responses and oxidative stress biomarkers while enhancing antioxidant defenses. The effects of SFN may be attributed to its mediation of Nrf2 activation, facilitating the restoration of immune balance and reducing oxidative stress, which leads to improvements in ASD symptoms (283). Although many studies demonstrated promising effects of SFN on ameliorating ASD-related symptoms, evidence on other mental and behavioral disorders, as well as cognitive functions related to SFN, is still insufficient.

Early-life intake of broccoli or its bioactive derivative SFN has demonstrated robust neuroprotective effects in children. Maternal intake of broccoli SFN may prevent behavioral abnormalities in the offspring via maternal immune activation (MIA), a known factor in the development of neuropsychiatric disorders (284). In an animal study, pregnant and lactating female mice were given food pellets enriched in glucoraphanin, a precursor of SFN, which demonstrated protective effects against MIA-induced cognitive deficits in mouse offspring (284). Juvenile offspring with glucoraphanin showed improved social interactions after MIA. This study also found that glucoraphanin prevented the loss of parvalbumin immunoreactivity in the medial prefrontal cortex, a key area implicated in cognitive function. Moreover, supplementation of SFN during the juvenile or adolescent stage helped prevent phencyclidine-induced cognitive deficits in schizophrenia in adulthood (285).

Studies have focused on the changes in the gut microbiome induced by maternal SFN treatment. It was found that maternal SFN significantly increased microbial *α*-diversity and *β*-diversity in the offspring (286). Significant changes at the genus level include an increased abundance of *Lactobacillus, Bifidobacterium, Ruminococcaceae UCG-014, and Intestinibacter*. In contrast, genera like *Oscillibacter*, *Enterorhabdus*, *Blautia*, and *Lachnospiraceae* were less abundant in the treatment group compared to the control. Additionally, they found changes in KEGG pathways and lower proinflammatory biomarkers in plasma, such as IL-6 and TNF-α, following SFN administration (286). In ASD children, SFN supplements enriched the beneficial bacteria *Bifidobacterium* and *Lactobacillales*, along with alleviating symptoms, indicating SFN may exert its neuroprotective effects through reshaping the gut microbiome profiles (282). Nonetheless, research focused on the impact of early-life or maternal treatment with SFN or cruciferous vegetable-related foods on brain development as well as the gut microbiome-driven mechanisms remains largely unexplored. Thus, further investigations are warranted to fully understand the underlying mechanisms and long-term impacts of SFN and its enriched vegetables on neurodevelopmental and behavioral disorders in the affected populations.

### 6.2 Tea polyphenol

Green tea is rich in polyphenols that significantly contribute to its health-promoting properties. The primary polyphenols in green tea include epigallocatechin-3-gallate (EGCG), epicatechin, and epigallocatechin. Numerous studies have demonstrated the beneficial effects of green tea on cancer, obesity, infections, and degenerative neurological diseases (287). The green tea polyphenols also show profound neuroprotective effects on brain health and behavior, mediated through their antioxidant, anti-inflammatory, and neuroprotective activities (288). Most of these health benefits can be attributed to their potent antioxidant and anti-inflammatory effects. Tea polyphenols (TPs) can influence the CNS through various mechanisms, including the interaction with the BBB, modulation of neurotransmitter systems, and effects on the cerebrovascular system (289, 290). Recent studies have shown that TPs exert their protective effects against neurodegenerative diseases by modulating the gut microbiome (289). The active metabolites produced by TPs and the enhanced gut microbiome diversity work synergistically to reduce neuronal damage and improve cell survival. This two-way mechanism involves both the direct and indirect neuroprotective effects that can inhibit inflammation and oxidative stress, leading to overall brain health (291). Therefore, TPs are considered bioactive compounds with neuroprotective and neuromodulatory impact (292).

Research in middle-aged and older adults has demonstrated the significant benefits of TPs on brain health (293). A clinical trial in elderly Japanese individuals found that higher consumption of green tea was associated with fewer depressive symptoms (294). Another study found a negative association between green tea consumption and cognitive impairment among the middle-aged Chinese population (295). This suggests that TPs may inhibit monoamine oxidase (MAO), resulting in increased monoamine levels in glial cells —a mechanism similar to that of antidepressants (296). Similarly, TP consumption showed antidepressant effects in mouse models with behavioral and depressive disorders (296, 297). In another mouse model, TPs improved recognition ability and reduced anxiety-like behaviors by suppressing TNF-*α* production (298). Previous studies found improvements in locomotor activity and spatial cognition learning ability in TP-treated animals (299, 300). Moreover, EGCG treatment has been shown to mitigate neurological damage in a rat model with ASD by regulating key gene expression associated with neurodevelopment (301). Similarly, TPs-derived flavanol catechin has demonstrated the ability to ameliorate behavioral, biochemical, neurological, and molecular impairments in ASD mouse models by modulating the nitric oxide pathway (302). In a human study, healthy young participants who consumed green tea for five weeks demonstrated improved reward learning and prevented depression-related symptoms (303). Similarly, a cross-sectional study found that higher consumption of green tea is associated with a lower prevalence of depression (304).

Early-life green tea consumption may render long-term beneficial effects on brain health later in life. One animal study has shown that offspring mice exposed to prenatal green tea consumption displayed faster sensory-motor reflex responses and notable stimulation effects (288). These beneficial effects further extended to adolescence, showing increased locomotor activity, decreased anxiety and fear, and improved memory and learning abilities. However, despite its potential benefits, green tea consumption is generally not recommended for young children due to its caffeine content and possible adverse effects. Future research should investigate the potential role of isolated tea polyphenols in pediatric neurodevelopment and behavioral correction, while considering safe and appropriate dosage guidelines.

TPs modulate the intestinal microbiota composition that benefits the host health. The intestinal microbiota can transform TPs into metabolites that exhibit significant neuroprotective effects (305). Specifically, tea catechins increased the proliferation of *Bifidobacterium* and *Lactobacillus/Enterococcus* groups and reduced the abundance of *Bacteroides*-*Prevotella*, *Clostridium histolyticum*, and *Eubacterium-Clostridium* groups. Additionally, the total SCFA concentrations in cells treated with EGCG were noticeably higher compared to the control (306, 307). In a mouse model with circadian rhythm disorder, TPs restored gut dysbiosis by increasing the abundance of *Akkermansia* and *Muribaculum* while decreasing *Desulfovibrio* (308). Another study found similar patterns of gut microbiome changes in response to TPs. TPs can inhibit harmful bacteria like *H. pylori, S. aureus, Bacteroides,* and *L. monocytogenes* while promoting beneficial probiotics such as *Bifidobacterium* and *Lactobacillus-Enterococcus* (309). Probiotics have been found to regulate abnormal brain activity by mitigating anxiety-and depression-like behaviors via the vagus nerve. *Lactobacillus* supplementation has been shown to reduce anxiety in mice by enhancing GABA receptor expression (309). TPs can prevent cognitive dysfunction by strengthening the intestinal barrier via targeting neuroinflammatory pathways (308).

### 6.3 Soy isoflavones

Soy foods have become a fundamental component in human diets, particularly among individuals following vegetarian and vegan lifestyles, due to their nutrient-dense protein content. Soybean products are rich in bioactive isoflavones, a class of phytoestrogens with a chemical structure similar to estrogen (310). There are three main isoflavone aglycones found in soybeans, including genistein, daidzein, and glycitein. Genistein is the most abundant isoflavone in soy products and has been well-studied due to its potent chemoprotective effects against various chronic human diseases (267, 310). Due to their structural similarity to estrogen, soy isoflavones can bind to estrogen receptor (ER), although their binding and activation capacity are significantly weaker compared to endogenous estrogen, 17-*β* estradiol (311). ERα and ERβ are the primary types of ER in mammals, among which ERβ is specifically involved in brain function (312). Interestingly, equol, a metabolite produced from isoflavones mediated by gut microbiota, may offer greater cognitive benefits due to its superior antioxidant properties, higher affinity to ERβ, longer bioavailability, and ability to enhance mitochondrial regulatory effects (313, 314). However, equol production depends on soy consumption and the presence of specific gut bacteria (315).

Studies have focused on the role of soy isoflavones in the prevention of breast cancer and other menopausal symptoms in postmenopausal women due to its phytoestrogenic effects (267). Moreover, estrogen is believed to have neuroprotective properties that play a significant role in cognitive function. There is a growing interest in exploring the beneficial effects of soy intake on cognitive functions due to its estrogen-like activity. Clinical studies have shown that soy isoflavone supplementation improves cognition and visual memory among postmenopausal women (316–318). Soy consumption has also been shown to improve memory, thinking, and behavior in Alzheimer’s patients through mechanisms such as reducing amyloid *β*, decreasing tau phosphorylation, and inhibiting the mitochondrial apoptotic pathway (319). Similarly, animal studies have concluded that dietary soy isoflavone supplementation improves spatial learning and memory skills in mice (320, 321).

Epidemiological studies support the important roles of estrogen in brain development and function. Therefore, early-life exposure to phytoestrogen-rich soy diets may influence early neurodevelopment in children. For example, fetuses and infants can access phytoestrogens through maternal routes via the placenta or breast milk (322). Phytoestrogens can bind to ER and activate estrogen-related signaling in the brain, which may influence the programming processes of the CNS (323). In a cohort study conducted on Chinese children, a moderate level of prenatal isoflavone exposure has been found to relate to a decreased risk of childhood neurobehavioral problems such as depression and anxiety. However, a high level of prenatal exposure negatively impacted behavioral health (324). Another study found that high prenatal isoflavone consumption decreased the risk of hyperactivity in Japanese children (325). This dose-dependent response seen in human cohort studies has been validated in animal studies, showing that a high level of prenatal soy exposure is related to an increased risk of offensive and depressed behaviors in mice (323, 326). Therefore, the dose and timing of soy isoflavone exposure may be critical in determining the outcomes of neurobehavioral development.

In animal studies, maternal exposure to soy isoflavone, daidzein, during pregnancy and lactation resulted in enhanced social affiliation and improved spatial memory associated with reduced ERα expression in the brain of the female offspring, suggesting that maternal daidzein may influence social and cognitive behaviors through, at least in part, altering ERα pathway (327). A large longitudinal study in children found that feeding soy formula during infancy was related to reduced female-typical play behavior (328). Moreover, a potential association between soy formula and autistic behaviors was found in humans (329). While the exact mechanisms remain unclear, some evidence suggests that the neuroprotective effects of isoflavones may be linked to their ability to regulate the expression of apoptosis-related genes, thereby inhibiting the mitochondrial apoptotic pathway in the brain (324, 330). Supportively, a rodent study found that the soy genistein intervention reduced apoptosis in hippocampal neurons (330).

Intensive studies have investigated the effects of soy isoflavones on the gut microbiome, bacterial metabolites, and host physiological responses. It was found that soy isoflavones can alter the composition and diversity of gut microbial communities. Additionally, soy isoflavones influence the production of bacterial metabolites such as SCFAs and neurotransmitter precursors, which play critical roles in maintaining gut and brain health (331, 332). Specifically, soy isoflavone treatment enhanced intestinal homeostasis, significantly reducing the levels of *Klebsiella*, *Pseudomonas*, *Acinetobacter*, *Escherichia-Shigella*, and *Staphylococcus*. Meanwhile, the relative abundance of beneficial bacteria, including *Bifidobacterium*, *Bacteroides*, *Dorea*, *Faecalibacterium*, and *Lactobacillus,* significantly increased (332). In another study, soy isoflavones significantly decreased *the Firmicutes species and lowered the Firmicutes-to-Bacteroidetes (F/B) ratio, while increasing the compositions of Proteobacteria and Actinobacteria* (333). Research indicates that memory ability is closely linked to gut microbiota composition, with *Bacteroidetes* and *Firmicutes* being the dominant phyla (334). Studies have shown that reduced microbiota diversity, decreased *Actinobacteria*, and an elevated F/B ratio were associated with cognitive decline, particularly in aging and neurodegenerative conditions (334). In this context, the observed reduction in the F/B ratio and microbial composition changes may correspond to improved memory retention following soy isoflavone treatment (333, 335). While extensive research has examined soy-induced gut microbiota changes and their role in aging-related cognitive decline, studies on its impact on brain development and memory function in children remain limited, underscoring a critical gap that requires further investigation.

### 6.4 Curcumin

Curcumin, a natural compound classified as a curcuminoid, has been used as a traditional Asian medicine due to its potent antioxidant and anti-inflammatory properties. Curcumin has been implicated in its roles in enhancing brain health and functions, validated in both *in vivo* and *in vitro* studies (336). Numerous studies indicate that curcumin could be a promising therapy for neurological disorders, as it can cross the BBB and exert its effects through Nrf2 activation and neuroinflammation suppression (337). Curcumin can also induce antidepressant effects by inhibiting the immobility period, increasing serotonin and dopamine levels, and inhibiting the MAO (338). Curcumin enhanced memory and learning abilities by boosting the cholinergic system and antioxidant activity (339). Furthermore, the beneficial effects of curcumin on long-term depression and anxiety have been linked to the upregulation of the brain-derived neurotrophic factor (BDNF) and the reduction of inflammatory factors in the brain (340). By reversing BDNF expression and extracellular signal-regulated kinase in the hippocampus, curcumin improved cognitive and behavioral parameters (341). Interestingly, curcumin also promoted the synthesis of PUFAs, such as DHA from its precursor, *α*-linolenic acid, and related enzymes in the liver and brain (342). Correspondingly, elevated DHA was associated with a reduction in anxiety-related behaviors in mice (343).

Beyond its impacts on neurological disorders and age-related neurodegenerative diseases, early-life curcumin supports brain development and hippocampal neurogenesis, enhancing neural plasticity and promoting neural repair (344). Specifically, maternal curcumin encourages the proliferation of embryonic cortical neural stem cells (NSCs) by activating ERK and p38 MAP kinase pathways, which are important for primary NSC proliferation (344). Besides maternal nutrition, curcumin exhibits significant neuroprotective benefits by mitigating the toxic effects of various environmental agents. For example, it counteracted Bisphenol-A induced neurogenesis impairment in developing rat brains by enhancing the proliferation and differentiation of NSCs through the Wnt/*β*-catenin signaling. Curcumin also alleviated mercury-induced neurodevelopmental deficits, restoring neurotransmitter balance and reducing anxiety in mouse offspring (345, 346). Similar neuroprotective effects of curcumin were observed when exposed to lead (347), valproic acid (348), fluoride (349), and alcohol (350) during pregnancy.

During the neonatal period, curcumin supplementation has been shown to improve autism-related symptoms in mouse models by increasing social interactions, reducing repetitive behaviors, and enhancing cognitive functions. Also, curcumin reversed the suppression of hippocampal neurogenesis (312). In the rat model of autism, neonatal curcumin supplementation can effectively improve birth weight and brain volume and restore several impaired parameters, including the depleted IFN-*γ*, serotonin, and glutamine (348). In another idiopathic mouse model of ASD, curcumin-enhanced α7-nicotinic acetylcholine receptors in CNS neurons reduced oxidative stress and alleviated ASD-like behavior, suggesting its potential as a novel pharmacological approach for ASD treatment (351). Studies regarding the effects of curcumin on ADHD are scarce. However, a preliminary behavioral study found that mice treated with curcumin exhibited less anxious and hyperactive behavior in the open field test and the social interaction test (352). Several clinical studies suggest that curcumin may be a beneficial antidepressant agent. However, these results remain inconclusive (353–356). Taken together, the neuroprotective effects of curcumin are promising, with significantly more research on its effects on depression and cognitive function than on its impact on ASD and ADHD.

Curcumin undergoes biotransformation through enzymes produced by enterocytes and hepatocytes, and is further metabolized by enzymes from the gut microbiota. These microbial enzymes can convert curcumin into bioactive derivatives, enhancing its stability, bioavailability, and therapeutic effects. Specifically, curcumin is metabolized by gut bacteria, such as *E. coli* and *Blautia* sp., producing bioactive derivatives, including tetrahydrocurcumin, demethylcurcumin, and ferulic acid (357). The pharmacological and pharmacokinetic properties of curcumin are mediated by several gut microbes, including *Bifidobacteria pseudocatenulatum, Enterococcus faecalis, Bifidobacteria longum, Lactobacillus acidophilus,* and *Lactobacillus casei* (358). On the other hand, curcumin administration improves gut microbial balance by increasing beneficial *Bifidobacteria* and *Lactobacilli* while reducing pathogenic *Prevotellaceae*, *Coriobacterales*, *Enterobacteria*, and *Enterococci* (359). In a mouse study, curcumin consumption led to a decrease in microbial diversity and richness, especially those bacteria triggering the onset of cancer and systemic diseases such as *Prevotellaceae, Bacteroidaceae, and Rikenellaceae* (357, 360). Curcumin and its gut microbiota-derived metabolites, particularly tetrahydrocurcumin, exhibit strong neuroprotective effects. They combat oxidative stress by enhancing antioxidant enzyme activity and modulating key pathways (PI3K/Akt, AMPK, Nrf2) involved in neuronal survival (357, 361). These findings highlight the crucial interplay between curcumin and the gut microbiota, emphasizing its role in modulating microbial composition and enhancing bioavailability through microbial metabolism. Given the neuroprotective effects and potential therapeutic applications of curcumin, future research should focus on optimizing its bioavailability and exploring its long-term impact on gut microbiota and neurological health.

### 6.5 Other bioactive phytochemicals

Research on bioactive phytochemicals is promising as emerging evidence highlights their potential to support neurodevelopment. In addition to the phytochemicals mentioned above, several other compounds, such as resveratrol, flavonoids, lutein, and carotenoids, play significant roles in neurodevelopment and cognitive function.

Resveratrol, commonly found in grapes, peanuts, and berries, is a natural polyphenol compound with antioxidant, anti-inflammatory, and neuroprotective properties. It can cross the BBB and regulate brain functions related to cognition, memory, and emotion, acting as a neuroprotective biofactor (362). Due to its antioxidant and anti-inflammatory effects, resveratrol has been explored in animal models of neurological diseases, implicating its beneficial effects in ASD (363). Clinical studies suggest that resveratrol, when used as an adjuvant treatment, improves ASD-related symptoms, such as irritability, hyperactivity, and non-compliance in children (364, 365). In animal models, resveratrol has demonstrated the ability to mitigate neuroinflammation in propionic acid-induced ASD mice, counteracting their neurological, sensory, behavioral, and molecular deficits. By modulating inflammatory cytokines and oxidative stress, resveratrol helps restore neuronal function and synaptic plasticity (366). Similarly, in the BTBR model of ASD, resveratrol exerted similar therapeutic effects by modifying the immune profiles (367). The pharmacological effects of resveratrol via gut microbiome modulation are also of interest. It reshaped the neurotoxin methylmercury-induced gut microbiome composition in mice. Specifically, resveratrol administration counteracted the changes caused by the toxin, restoring the *Firmicutes*/*Bacteroidetes* ratio and increasing *Lactobacillus* and *Muribaculaceae_unclassified* while reducing the abundance of the toxin-upregulated genera (368). Interestingly, resveratrol and gut microbiota have a mutual influence. Resveratrol alters microbial composition while being metabolized by certain bacteria. *Bifidobacteria infantis* and *Lactobacillus acidophilus* contribute to piceid production from resveratrol (369). Additionally, resveratrol enhances gut microbiota diversity by inhibiting *Enterococcus faecalis* and promoting the growth of *Lactobacillus* and *Bifidobacterium* (370). In addition to the changes in gut microbiome composition, resveratrol is also involved in the regulation of glucagon-like peptide 1 and the 5-hydroxytryptamine level, ultimately impacting brain function via the GBA (371).

Flavonoids, abundant in fruits, vegetables, and tea, support neuroprotection by reducing neuroinflammation and promoting synaptic plasticity. Their effects on the brain are attributed to their ability to protect neurons, enhance neuronal function, and promote neurogenesis. Evidence indicates that flavonoids exert neuroprotective actions by modulating key intracellular signaling pathways involved in neuronal survival, differentiation, and memory formation (372). Preclinical studies suggest that flavonoids possess antidepressant properties by reversing depression-related deficits through their antioxidant activity, neurotransmitter modulation, increased BDNF levels, and regulation of key enzyme activities (373). One intervention trial in children demonstrated positive mood effects following the consumption of a flavonoid-rich blueberry drink, likely due to the increased cerebral blood flow and inhibited MAO activity (374). A similar intervention trial in young adults found improvements in depression and gut microbiome composition (375). Beyond depression, flavonoid administration has been shown to improve ASD-related features, including socialization deficits and repetitive or stereotypic behaviors in both animal and human studies (376). Although promising, more human studies are needed to confirm the efficacy of flavonoids as a therapeutic approach. Besides, flavonoids alter gut microbiota by replacing harmful pathogens with beneficial bacteria and promoting the production of SCFAs (377). In an intervention trial of depression, flavonoids increased *Bacteroidetes* and *Actinobacteria* phyla, as well as *Lachnospiraceae*, *Bifidobacteriaceae*, and *Akkermansiaceae* families. At the genus level, genera like *Lactobacillus*, *Alistipes*, *Roseburia*, *Akkermansia*, *Bifidobacterium*, and *Collinsella* became more abundant, while *Faecalibacterium*, *Streptococcus*, and *Eubacterium_g23* showed lower abundance (375). They also found a positive correlation between the BDNF level and the Lachnospiraceae family. These changes suggest that flavonoid treatment altered gut microbiota composition, which may play a role in its therapeutic effects on depression (375).

Carotenoids, a class of naturally occurring pigments in fruits and vegetables, play essential roles in brain health and cognitive function. Lutein and zeaxanthin, which are particularly abundant in dark leafy greens, can cross the BBB and accumulate in the brain and retina (378). In the developing brain, lutein constitutes a significantly larger proportion of total carotenoids compared to adults, underscoring its importance in early cognitive function, visual processing, and overall brain maturation during childhood (379). Chronic inflammation is associated with reduced BDNF expression, which impairs neuroplasticity and cognitive function. The anti-inflammatory capability of lutein can increase the BDNF (380). In adults, lutein and zeaxanthin levels are positively related to better cognitive function. Recent studies have shown that early consumption of lutein and zeaxanthin may improve childhood receptive vocabulary and mid-childhood executive function (381). One meta-analysis found that carotenoids are effective in improving depressive symptoms, with animal studies supporting this by showing that zeaxanthin treatment reduced the levels of IL-6, IL-1*β*, and TNF-*α* in the hippocampus (382). Evidence of the interaction between carotenoids and the gut microbiome is still lacking. One study showed that all four carotenoids, including β-carotene, lutein, lycopene, and astaxanthin, increased the abundance of *Roseburia* and *Parasutterella* and inhibited the abundance of *Collinsella* (383). Moreover, carotenoids with different structures have distinct impacts on the gut microbiome. For example, β-Carotene notably increased SCFA production by promoting the growth of SCFA-producing bacteria. Xanthophylls such as lutein and zeaxanthin have a more significant impact on the gut microbiome than carotenes (383).

Overall, these phytochemicals play essential roles in brain function, as shown in Table 2. Their neuroprotective properties contribute to cognitive function, emotional regulation, and neurodevelopment, making them promise for supporting brain health and alleviating neurological disorders. The effects of many other dietary compounds remain underexplored, particularly in the context of their interactions with the GBA. Further research is needed to identify and characterize these compounds and elucidate their mechanisms of action in brain and microbiome health.

## 7 Limitation

While this review highlights the extensive evidence about the association between early-life nutrition, the gut microbiome, and neurodevelopment, several limitations should be acknowledged. First, although maternal nutrition and childhood behaviors are well studied, paternal influences, including paternal age, diet, health status, stress, and microbiome contributions, were not explored in depth in this review. Emerging research suggests that paternal factors can also impact offspring neurodevelopment through epigenetic and microbial pathways, and their exclusion may give an incomplete picture of early-life factors of brain health (13, 384, 385). Moreover, while findings from animal studies, clinical trials, and epidemiological research suggest a compelling role for dietary interventions in supporting neurodevelopment and gut health, the evidence remains mixed, particularly in conditions such as ADHD. Inconsistent results may arise from methodological variability, differences in microbiome analysis, heterogeneous age groups, and the presence of comorbidities. These factors highlight the need for more standardized, large-scale, and longitudinal studies to better understand causal relationships.

## 8 Conclusion

The interplay between nutrition, the gut microbiome, and brain development plays a fundamental role in influencing child behavior and cognitive function. This review emphasizes the bidirectional communication between the gut and brain, highlighting the impacts of early-life nutrition on neurodevelopmental outcomes. Dietary patterns, as well as bioactive components in food such as tea polyphenols, broccoli-derived sulforaphane, curcumin, and soy isoflavones, have shown promising neuroprotective effects by modulating oxidative stress, neuroinflammation, and gut microbiota composition. Conversely, disruptions in microbial balance, often influenced by unhealthy dietary patterns and deficits in specific nutrients, have been linked to neurodevelopmental disorders, including ASD, ADHD, and anxiety. Clinically, current results support the potential of early-life nutrition and gut microbiome modulation to promote optimal brain development and mitigate behavioral disorders in children. A holistic approach that integrates maternal and early-life nutrition, dietary patterns, and bioactive nutrient intake may offer a promising strategy for promoting lifelong brain health, cognitive well-being, and a healthy gut system.

## Glossary

ADHD: Attention Deficit Hyperactivity Disorder
ASD: Autism Spectrum Disorder
ANS: Autonomic Nervous System
BBB: Blood–Brain Barrier
CNS: Central Nervous System
CD: Conduct Disorder
DASH: Dietary Approaches to Stop Hypertension
DHA: Docosahexaenoic Acid
EPA: Eicosapentaenoic Acid
ENS: Enteric Nervous System
EGCG: Epigallocatechin-3-Gallate
FGR: Fetal Growth Restriction
FMT: Fecal Microbiome Transplantation
FSIQ: Full-Scale IQ
GABA: *γ*-Aminobutyric Acid
GF: Germ-Free
GI: Gastrointestinal
GBA: Gut-Brain Axis
HFD: High-Fat Diet
HPA: Hypothalamic Pituitary Adrenal
HMOs: Human Milk Oligosaccharides
IQ: Intelligence Quotient
IFN-γ: Interferon-Gamma
KD: Ketogenic Diet
MD: Mediterranean Diet
MHD: Mental Health Disorders
MAO: Monoamine Oxidase
mTOR: Mammalian Target of the Rapamycin
Nrf2: Nuclear Factor Erythroid 2-Related Factor 2
NSCs: Neural Stem Cells
ODD: Oppositional Defiant Disorder
PUFAs: Polyunsaturated Fatty Acids
PD: Plant-Based Diet
RA: Retinoic Acid
SCFAs: Short-Chain Fatty Acids
SHR: Spontaneously Hypertensive Rats
SFN: Sulforaphane
TP: Tea Polyphenol
TNF-*α*: Tumor Necrosis Factor-Alpha
